# The Role of Insulin Resistance and Protein O-GlcNAcylation in Neurodegeneration

**DOI:** 10.3389/fnins.2019.00473

**Published:** 2019-05-09

**Authors:** Suraiya A. Ansari, Bright Starling Emerald

**Affiliations:** ^1^Department of Biochemistry, College of Medicine and Health Sciences, UAE University, Al Ain, United Arab Emirates; ^2^Department of Anatomy, College of Medicine and Health Sciences, UAE University, Al Ain, United Arab Emirates

**Keywords:** insulin resistance, brain insulin uptake, Alzhemier’s disease, O-GlcNAc cycling, neurodegeneration

## Abstract

Metabolic syndrome including obesity and type 2 diabetes is increasing at an alarming rate worldwide. Similarly, there has been an increase in the cases of neurodegenerative diseases such as Alzheimer’s disease (AD) possibility due to increase in elderly population in the past few decades. Both, metabolic diseases and AD have one common feature that is insulin resistance. Recent studies suggest a link between the regulatory functions of insulin in the brain and AD. Hypoglycemia, a characteristic feature of AD may be a result of impaired insulin signaling in the affected regions of the brain. O-GlcNAcylation is a post-translational protein modification, the levels of which are dependent on the availability of glucose inside the cells. Hyperphosphorylation of Tau is a major molecular feature, which leads to its aggregation and neurotoxicity in AD. In addition, impaired processing of Amyloid precursor protein (APP) leading to toxic amyloid β (Aβ) aggregation is also implicated in the pathogenesis of AD. Both APP and Tau are also found to be O-GlcNAcylated. Reduced O-GlcNAcylation of APP and Tau due to hypoglycemia is found to be associated with their pathological features in AD brain. Recent studies have also identified perturbed O-GlcNAcylation/phosphorylation of several other proteins important for normal neuronal function, which may be contributing to the neuropathological development in AD. Herein, we discuss about the uptake and distribution of insulin inside the brain, brain insulin signaling and insulin resistance as well as its relation to neurodegenerative diseases with a special focus on protein O-GlcNAcylation and its potential role in the treatment of AD.

## Introduction

Diabetes mellitus (DM) is an endocrine disorder affecting millions of people worldwide. DM is classified into two major forms, type 1 diabetes mellitus (T1DM) and type 2 diabetes mellitus (T2DM). T2DM is mainly due to insulin resistance and is one of the key components of the metabolic syndrome that also contributes to obesity. T2DM is also the most common form of DM, accounting for more than 90% of all types of DM, and thus taking up most of the funds available for the management of patients with this condition. Unfortunately, the prevalence of T2DM has reached an epidemic state in the last few decades, accounting for more than 425 million diabetics around the globe. It is projected that there will 629 million people with diabetes by the year 2045 (IDF Diabetes Atlas, 2017).

Central nervous system controls important physiological and metabolic processes such as feeding, acquisition of energy and expenditure, regulation of body weight and glucose homeostasis, (Schwartz and Porte, 2005; Prodi and Obici, 2006). Although a link between diabetes, glucose homeostasis and brain has been established by classical experiments almost a century ago using experiments in dogs, where impairment of the fourth ventricle resulted in marked glycosuria (Bernard, 1854), the brain was considered as an insulin insensitive organ for decades because passive diffusion into the brain is limited by the blood-brain barrier (BBB). Experiments using radioactive phosphorus and radiolabeled 131I-insulin in the early 60s have established that at least some parts of the brain are sensitive to insulin and insulin may have regulatory roles in the brain (Chowers et al., 1966). According to more recent studies, insulin in addition to its major regulatory roles in the peripheral tissues such as liver, muscle, and adipose tissue has emerged as a major regulatory molecule in the central nervous system. It has been suggested that hormones such as insulin circulate through the central nervous system and integrate the feeding behavior, autonomic outflow and metabolism so that a homeostasis is maintained between energy metabolism and storage of excess energy (Schwartz and Porte, 2005). Studies have established the presence of insulin like peptides (IGF1/2) and insulin receptors (IR) in the brain and shown to be involved in the regulation of different metabolic processes such as feeding and cognition (Schechter et al., 1990, 1994, 1996). The IRs of the brain were shown to be similar in structure to the ones present in peripheral tissues but there are differences in their distribution in different cell types which is more in the neurons compared to the glial cells (Schwartz et al., 1992; Wozniak et al., 1993). Furthermore, although insulin insensitive glucose transporters, Glut1 and Glut3 are mainly responsible for glucose uptake inside the brain, it is also shown that, in the neurons of the hypothalamus, glucose is transported via insulin sensitive Glut4 transporter (McEwen and Reagan, 2004; Ren et al., 2014).

Although, it is well accepted that T2DM is predominantly a genetic disease, the drastic increase in the worldwide prevalence of T2DM highlights the changes in regulatory mechanism that happens at multiple levels of metabolism. Insulin, the primary hormone which was isolated almost a century ago (Banting et al., 1922) is secreted by the β-cells of the islets, exerts its function through different peripheral tissues and prevents postprandial hyperglycemia, and maintains euglycaemic levels. Through its action in the skeletal muscle, it promotes the uptake of glucose from the blood stream and inside the liver, it prevents the production of glucose by preventing gluconeogenesis and glycogenolysis and promoting glycogen synthesis (DeFronzo and Ferrannini, 1991). Studies aimed at the estimation of insulin in both human and rodent brain have noticed a higher concentration of insulin in brain extract compared to that of the plasma (Havrankova et al., 1978). This abundance of insulin in brain suggests a possible important regulatory role for it in the brain. In this review, we discuss about insulin signaling in the brain and the role of insulin resistance in neurodegenerative diseases such as Alzheimer’s disease (AD) with a particular focus on the association of protein O-GlcNAcylation with impaired glucose utilization possibly due to insulin resistance and the role of O-GlcNAcylation on the pathology of AD.

## Distribution of Insulin in the Brain

Insulin is a peptide hormone of 51-amino acid, which has a helical native structure and a molecular weight of about 6,000 Da (Jimenez et al., 2002). Its entry into the brain is limited by the BBB and the precise mechanisms of how and where insulin enters the brain remains unclear. Recent studies have suggested that the entry of insulin into the brain parenchyma can occur directly via the median eminence (ME), indirectly through the cerebrospinal fluid (CSF) or through brain interstitial fluid (ISF). The possibility that insulin may reach the brain through ME is based on the fact that some part of the brain such as circumventricular organs (CVOs) are without BBB and are with fenestrated capillaries which make the passage connection between the blood circulation and the underlying brain parenchyma (Siso et al., 2010). In fact, administration of radioactive insulin has been shown to reach the arcuate nucleus (ARH) of the hypothalamus through ME (Corp et al., 1986). Increase in the levels of insulin led to the activation of AKT signaling (insulin receptor activation) and c-Fos signaling (neuronal activation) in the ARH. This is important as defects in AKT and c-Fos signaling contributes to insulin resistance which leads to T2D and obesity (Olson et al., 1993; Koch et al., 2008; Williams et al., 2010; Clegg et al., 2011). Although the tight junctions between the tanycytes may restrict the movement of insulin and other hormones, they may reach other parts of the brain through ISF. This possibility is supported by the fact that the quantitation of ARH ISF showed a low level of insulin upon fasting which increased after feeding or peripheral administration of insulin (Gerozissis et al., 1997; Langlet et al., 2013) and this effect was very rapid suggestive of diffusion through ME. CSF, which is produced by the choroid plexus in the brain reaches Virchow-Robin space through the third and fourth ventricles and the microvasculature helps in the transfer of insulin and other hormones from blood circulation to CSF (Iliff et al., 2012). However, the significance of these modes of entry of insulin to the brain is still not well understood. Insulin binds to IR, which is a heterotetrameric receptor (2 α subunits and 2 β-subunits) (Boucher et al., 2014). In humans, the IR gene codes for two isoforms A and B and the uniformly expressed isoform A is expressed predominantly in the brain (Frasca et al., 1999). Although it shows variations, it is expressed in the hypothalamus, olfactory and limbic areas, neocortex, hippocampus, basal ganglia, cerebellum and choroid plexus (Hill et al., 1986). It was also shown that highest IR expression was seen in the ARH (Plum et al., 2005). Binding of insulin to IR results in its autophosphorylation and signals through the insulin receptor substrate proteins (IRS) which leads to the activation of P13K/AKT signaling pathway, an important member of the complex network mediating insulin signaling (Rojas et al., 2003). In this pathway, P13K a lipid kinase that acts as a heterodimeric enzyme, phosphorylates the D-3 position of the inositol ring in phosphoinositides (Whitman et al., 1988; Carpenter et al., 1990; Stephens et al., 1991) and when insulin secretion is stimulated by the presence of glucose in the circulation, this signaling cascade that phosphorylates the serine-threonine kinase, AKT is activated. AKT phosphorylation then triggers the uptake and metabolism of glucose and coordinates neuromodulatory gene transcription (Kan et al., 1994; Hill et al., 1999). Insulin signaling is also negatively regulated by protein tyrosine phosphatases (PTPs), T cell protein tyrosine phosphatase (TCPTP) and suppressor of cytokine signaling 3 (SOCS3) (Wunderlich et al., 2013; Zhang et al., 2015). In addition, the other members of insulin family of peptides such as insulin like growth factor 1 and 2 (IGF1/2) also play important roles in the control of energy metabolism in the brain and neuronal plasticity (Fernandez and Torres-Aleman, 2012).

## Insulin Signaling in the Brain

The main target of insulin signaling in the brain is shown to be the hypothalamus (Heni et al., 2015). Functional analysis have shown several regions in the hypothalamus such as the ARH, the ventromedial nucleus (VMH), lateral hypothalamic area (LHA), dorsomedial nucleus of the hypothalamus (DMH) and paraventricular nucleus of the hypothalamus (PVH) respond to the changes in the level of insulin (Porter and Bokil, 1997; Qiu et al., 2014). Specifically, in the hypothalamus, the response to the insulin level had been shown to be with the two group of neurons, the POMC [α-melanocyte-stimulating hormone (MSH) precursor] which is anorectic in function and the agouti-related peptide (AgRP)/neuropeptide Y (NPY)-neuropeptide expressing neurons which are orexigenic (Varela and Horvath, 2012). While activation of AgRP neurons promotes feeding and weight gain, the activation of POMC neurons increase satiation, increase energy expenditure and attenuate weight gain (Xu and Xie, 2016). Insulin signaling in the brain has been shown to inhibit AgRP/NPy neurons while it activates POMC neurons (Varela and Horvath, 2012; Qiu et al., 2014; Roh et al., 2016). It was also suggested that from these neurons through the neuronal extensions insulin may be reaching the different regions of the hypothalamus such as the PVH, VMH, DMH, lateral hypothalamus (LH), amygdala, bed nucleus of stria terminalis, parabrachial nucleus and the dorsal vagal complex (Wang et al., 2015). Apart from these, insulin was also shown to act on the SF1 neurons of the VMH and in the dopaminergic neurons of the midbrain and in the higher cortical regions (Guthoff et al., 2010; Klockener et al., 2011; Konner et al., 2011).

## The Effects of Systemic Insulin Resistance in the Brain

Insulin resistance, the main contributor of T2D and obesity results from that fact that although there is high levels of insulin present in the circulation, the response to this is defective and brain is not an exception to this (Zhang et al., 2015). Interestingly, it has been shown that the effect of insulin in the brain is completely lost in the case of obesity and is restored with weight loss (Tschritter et al., 2006; Tuulari et al., 2013). Among the contribution of different factors in the development of insulin resistance, the intake of high fat diet has been suggested to reduce the sensitivity of hypothalamic insulin (Clegg et al., 2011). With the increase in the availability of nutrient rich food, this may be one reason which could contribute to the increase in obesity worldwide. It has been shown that saturated fatty acid such as palmitate or stearate can cross the BBB and can activate inflammatory signaling in the hypothalamus resulting in insulin resistance (Kleinridders et al., 2009). An increase in the levels of the negative regulators of insulin signaling in the hypothalamus such as TCPTP, PTP1B, and SOCS3 also have been shown and these factors also increase as a result of obesity induced inflammation (Loh et al., 2011; Zhang et al., 2015). Another factor which can contribute to insulin resistance is the decreased BBB permeability resulting in defective signaling (Kern et al., 2006; Hsu and Kanoski, 2014). Diet induced obesity also results from gliosis which makes both POMC and AgRP neurons insensitive to the peripheral insulin (Horvath et al., 2010; Dorfman and Thaler, 2015). So the main roles of insulin in the brain, regulation of body homeostasis and balancing of nutrient intake and energy expenditure are all altered in insulin resistance.

## Insulin Resistance in Neurodegeneration

An association of T2DM and neurodegenerative diseases such as AD has also been described (Holscher, 2011; Chen et al., 2014). AD is a neurodegenerative disease characterized by progressive dementia and loss of cognitive abilities. The incidence of both T2DM and AD increase with age and various epidemiological studies have shown an increased risk of dementia and neurodegeneration in people with T2DM (Mushtaq et al., 2014; Alam et al., 2016). Further, there are several common biological mechanisms associated with the pathogenesis of T2DM and AD such as insulin resistance and impaired glucose metabolism, Aβ plaque formation in the brain and increased oxidative stress (Zhao and Townsend, 2009; Mushtaq et al., 2014). Interestingly, the disruption of normal glucose metabolism in the affected region of AD brain is shown to promote Aβ aggregation that is associated with neurodegeneration observed in AD (Li et al., 2007; Zhao and Townsend, 2009).

Although vascular complications of diabetes may also be responsible for the development of neurodegenerative diseases in advanced age, a direct contribution of impaired insulin signaling pathway has been implicated repeatedly (Steen et al., 2005; Zhao and Townsend, 2009; Ma et al., 2015). Hyperglycemia or chronically elevated blood glucose level is associated with insulin resistance and development of T2DM. At the molecular level, the hallmarks of insulin resistance in major insulin target tissues such as liver, muscle and adipose tissue include defects in the expression and phosphorylation of many effector molecules of insulin signaling pathway such as insulin receptor (IR), IRS1/2, PI3K and AKT (PKB), reduced expression of Glut4, and increased hepatic gluconeogenesis and lipogenesis (Mlinar et al., 2007; Samuel and Shulman, 2012).

As explained above, IR is expressed throughout the brain and in larger part, the uptake of glucose takes place with the help of glucose transporters, Glut1 or Glut3 in an insulin independent manner, the dependence on insulin for glucose uptake has been observed in specific areas of brain (Vannucci et al., 1998; Reno et al., 2017) through insulin dependent glucose transporter, Glut4. Therefore, a defect in brain insulin signaling may lead to reduced glucose uptake. Earlier works, which tried to determine the concentrations of insulin as well as IR in aging and AD brains, have shown by immunohistochemical staining in the neocortex of postmortem brains that the levels of insulin and connecting peptide (c-peptide) of insulin are reduced in both aging and AD brains. Furthermore, the levels of IR was decreased in the aging brain whereas it increased in AD brain compared to age matched controls (Frolich et al., 1998). Relatively a recent study, with real time PCR technique as well as immunostaining has shown reductions in the expression of IR, IGF1 and IGF2 polypeptides and their receptors in the AD brains. This correlated with increased levels of amyloid protein precursor (APP), glial fibrillary acidic protein, and the IBA1/AIF1 microglial mRNA transcripts (Rivera et al., 2005; Steen et al., 2005). As there is reduced levels of IR, along with decreased production of growth factors and their receptors (IGF1/2) and if neurons are dependent on the local production of these growth factors then a reduced production may lead to neuronal cell death which is what is seen in case of AD (Steen et al., 2005). Further on this, the role of inhibitory serine phosphorylation on IRS1 in the case of insulin resistance has been reported in most of the insulin target tissues including brain (Aguirre et al., 2002; Gual et al., 2005; Lerner-Marmarosh et al., 2005; Draznin, 2006; Barone et al., 2016). It is well established that Tyrosine phosphorylation of IRS1 during insulin signaling is responsible for signal activating function of IRS1 through its association with downstream effector, PI3K whereas ser307, S612, and S632 (ser312, S616, and/or S636 in human) of IRS1 is inhibitory to downstream insulin signal transduction (Aguirre et al., 2002; Draznin, 2006; Talbot et al., 2012). Consistent with this, increased ser307 phosphorylation is reported in insulin resistance of insulin target tissues such as skeletal muscle as well as in AD brain (Bandyopadhyay et al., 2005; Lerner-Marmarosh et al., 2005; Talbot et al., 2012; Barone et al., 2016). Interestingly, increased phosphorylation of inhibitory ser616 and ser636 of IRS1 were reported in AD brains even without peripheral insulin resistance (Talbot et al., 2012). Furthermore, brain insulin resistance is also suggested a risk factor for cognitive impairment (Ma et al., 2015). Although a clear molecular understanding behind insulin resistance and cognitive impairment is not known, various mechanisms such as defects in the neuronal plasticity and increased inflammation due to altered PI3K/AKT/GSK-3 signaling have been described based on animal models and epidemiological data (Calvo-Ochoa and Arias, 2015; Neergaard et al., 2017). Overall, these studies suggest a clear correlation between insulin receptor signaling and insulin resistance in the development of AD.

## Role of Protein O-GlcNAcylation in Insulin Signaling

The role of glucose responsive protein O-GlcNAcylation has emerged as an important player in insulin signaling as it has been reported that many effector molecules of insulin signaling pathway are also O-GlcNAcylated, often in a reciprocal manner to phosphorylation on specific serine residues (Love and Hanover, 2005; Ma and Hart, 2013, 2014; Myslicki et al., 2014). O-GlcNAcylation is a post-translational protein modification on the serine/threonine amino acid of various proteins through the transfer of single sugar molecule, β-*N*-acetylglucosamine from the substrate UDP-GlcNAc. Protein O-GlcNAcylation was first discovered by Gerald Hart and colleagues in early 1980s as a dynamic protein mono-glycosylation present on nucleocytoplasmic proteins. Since then, thousands of proteins have been identified to be modified through this protein modification (Nandi et al., 2006; Hahne et al., 2013; Li et al., 2016). UDP-GlcNAc, the substrate needed for protein O-GlcNAcylation is produced through a metabolic pathway known as Hexosamine Biosynthesis Pathway (HBP) which is activated after the entry of glucose into the cells, where a small percentage of this glucose (2–5%) is shoveled into HBP resulting in the generation of UDP-GlcNAc through several steps (Love and Hanover, 2005; Hanover et al., 2010). The dynamic addition or removal of O-GlcNAc on serine/threonine amino acids of target proteins is achieved with the help of a pair of enzymes, O-GlcNAc-transferase (OGT) and O-GlcNAase (OGA). OGT attaches O-GlcNAc moiety provided by UDP-GlcNAc on to the target proteins whereas OGA removes it (Iyer and Hart, 2003; Hanover et al., 2010; Bond and Hanover, 2015; Eustice et al., 2017). Hyperglycemia is shown to increase the total levels of O-GlcNAc inside the cells as has been shown in several previous studies (Copeland et al., 2008). Interestingly, it has been reported that almost all of the major players of insulin signaling pathway such as IRS1, PI3K, PDK1, AKT, and FOXO1 are also O-GlcNAcylated often reciprocal to the phosphorylation sites on these proteins and thereby regulate insulin signaling through positive/negative feedback. Therefore, chronic elevation of O-GlcNAc could be considered a mechanism in the development of insulin resistance at least in part through O-GlcNAcylation of PI3K or AKT on stimulatory serine residues (Whelan et al., 2010; Ma and Hart, 2013). Toward this end, studies using animal models and cell culture experiments have shown that increasing O-GlcNAc levels due to either genetic or pharmacological inhibition of OGA activity leads to development of symptoms of insulin resistance and T2DM (Vosseller et al., 2002; Arias et al., 2004; Park et al., 2005; Keembiyehetty et al., 2015). However, Inhibition of O-GlcNAcase in 3T3-L1 adipocytes using a potent inhibitor on OGA did not induce insulin resistance (Macauley et al., 2010) suggesting a more complex relationship between phosphorylation and O-GlcNAcylation rather than just a yin-yang mechanism. Interestingly, inhibitory serine residues of IRS1 can also be O-GlcNAcylated (Jahangir et al., 2014) leading to the possibility of hypoglycemia mediated reduced O-GlcNAcylation on inhibitory serine residues of IRS1 that may result in its increased phosphorylation and development of impaired insulin signaling in AD brain.

This suggests the possibility of a harmonious balance between O-GlcNAcylation and phosphorylation at the physiological levels is necessary for proper functioning of insulin pathway and an impairment in this homeostasis might be responsible for the development of pathology (Yang and Qian, 2017).

## Link Between Brain Insulin Resistance, O-GlcNAcylation and Neurodegeneration

**FIGURE 1:**
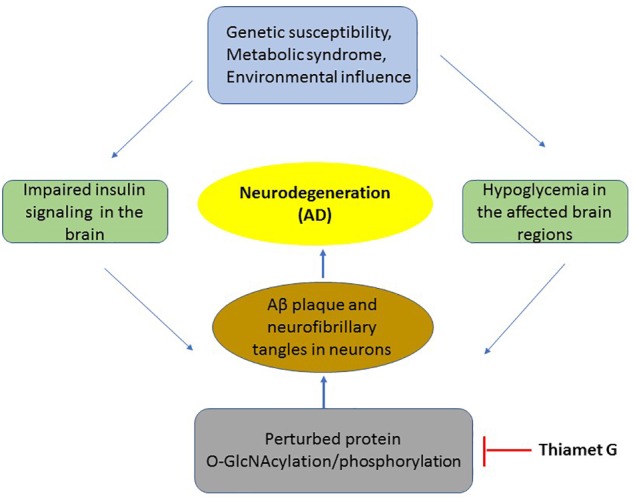
The mechanism of insulin resistance and hypoglycaemia on pathology of neurons in AD. A flowchart of key events occurring in the development of AD which includes metabolic syndrome or disease susceptibility to neurodegeneration which results in impaired insulin signaling and hypoglycaemia leading to reduced O-GlcNAcylation of several proteins including APP and Tau. This results in the production of toxic Aβ aggregation and neurofibrillary tangles leading in neuronal death. Thiamet G by inhibition of OGA increases O-GlcNAcylation thus blocking downstream pathological events of AD.

Hypoglycemia is one of the common features of many neurodegenerative diseases including AD (Hoyer, 2004). Several studies have identified insufficient glucose uptake and utilization in the affected regions of AD brain (Pedersen et al., 1999; Daulatzai, 2017). Insulin resistance in the brain is observed due to defects in insulin receptors signaling (Kuljis and Salkovic-Petrisic, 2011) and decreased levels of brain glucose transporters, Glut1 and Glut3 (Liu et al., 2008; Szablewski, 2017) which is also observed in AD brain. Furthermore, using postmortem AD brains, Talbot et al show that both IR and IGF1 responsiveness and downstream signaling through these receptors were significantly reduced in AD brains (Talbot et al., 2012). Therefore, although insulin has several functions in the brain, a defect in the effectors of insulin signaling pathway as observed in peripheral insulin resistance may also be responsible for brain insulin resistance (Candeias et al., 2012; Duarte et al., 2012; Chen and Zhong, 2013). Hypoglycemia in AD brain leads to decreased protein O-GlcNAcylation. Several studies have recently associated decreased O-GlcNAcylation to the pathogenesis of AD using both *in-vitro* and *in-vivo* experiments (Liu Y. et al., 2009; Gong et al., 2016; Pinho et al., 2018). The common theme that had emerged from these studies suggests that decreased O-GlcNAcylation of beta-amyloid precursor protein (APP) and Tau, two main culprits associated with neurodegeneration in Alzheimer’s are associated with increased phosphorylation thus leading to classical Aβ plaque formation and Tau aggregation (Dias and Hart, 2007). The initial studies led by Robertson et al. (2004) and later by Liu F. et al. (2009) showed a reciprocal relationship between phosphorylation and O-GlcNAcylation on Tau protein suggesting that changes in Tau glycosylation may influence its phosphorylation state (Robertson et al., 2004). The levels of total O-GlcNAc were found to be reduced in AD brain, which negatively correlated with phosphorylation of Tau (Liu F. et al., 2009). These results suggested that impaired glucose metabolism leading to reduced O-GlcNAcylation of Tau results in its hyperphosphorylation [3–4 folds more phosphate than normal Tau (Liu et al., 2004)] and neurofibrillary degeneration in AD. Similarly, APP had been found be O-GlcNAcylated (Griffith et al., 1995) and that this plays an important role in its processing (Jacobsen and Iverfeldt, 2011; Chun et al., 2015). The accumulation of hydrophobic amyloid-beta (Aβ) peptide is a hallmark feature of AD. APP is processed through two proteolytic cleavage pathways termed as non-amyloidogenic pathway and amyloidogenic pathway where former is favored in normal brain whereas later pathway is found to be more active in AD brain leading to increased formation of pathogenic Aβ peptide. A study by Jacobsen et al showed that increasing the levels of total O-GlcNAc through PuGNAc to inhibit the function of OGA resulted in an increase in the level of O-GlcNAcylated APP, with increased secretion of sAPPα and decreased Aβ secretion (Jacobsen and Iverfeldt, 2011). Furthermore, Yuzwa et al., 2012 used a hemizygous JNPL3 tau mouse model (which express mutant human P301L tau whose expression is roughly equivalent to that of endogenous mouse tau and these animals undergo progressive neurodegeneration) and showed that increasing the levels of O-GlcNAc stabilized Tau aggregation and slowed down neurodegeneration. Later studies further confirmed the effect of OGA inhibition on preventing Tau aggregation and amelioration of pathological features in mouse model of tauopathy (Graham et al., 2014). Similarly, beneficial effect of OGA inhibition on the Aβ plaque formation and memory impairment has been observed in a mouse model of AD (Kim et al., 2013). Therefore, there is a significant link between hypoglycaemia and AD where protein O-GlcNAcylation plays an important role in the production of toxic APP and Tau aggregation due to a decrease in O-GlcNAcylation of these proteins (Figure 1). Recent studies have identified several other proteins which belong to important functional categories such as memory associated proteins, cytoskeleton and synaptic proteins with altered O-GlcNAc levels in the postmortem AD brain (Wang et al., 2017). Wang et al show that among the altered O-GlcNAcylation in AD brains, proteins of particular interest which showed reduced O-GlcNAcylation in AD brain are ANK3 (ankyrin-3) and SYNPO (synaptopodin) which are involved in membrane integrity/axon polarity and synaptic plasticity, respectively, (Wang et al., 2017). In another recent study, using a triple transgenic mouse model of AD (3 × Tg-AD) and using an antibody specific for O-GlcNAc sugar to enrich all proteins O-GlcNAcylated in control and AD brains, Tramutola et al have identified several proteins which are important for neuronal function including structural proteins such as α tubulin, NF-L (neurofilament light chain) and energy metabolism such as Gapdh, Eno1, and Madh which are important enzymes of glycolysis and Kreb cycle (Tramutola et al., 2018).

Therefore, we cannot exclude the possibility of the role played by altered O-GlcNAcylation of these proteins as discussed above in the pathogenesis of AD and further studies are needed to functionally characterize the role of protein O-GlcNAcylation in relation to phosphorylation on these proteins and their relevance to AD.

## Conclusion

It is clear that insulin, either through its uptake from systemic blood circulation or through local production plays significant roles in glucose homeostasis and energy metabolism in the brain. Insulin resistance, as observed in case of metabolic syndrome and T2DM or in neurodegenerative disease such as AD plays an important role in the pathogenesis of these diseases. Protein O-GlcNAcylation has emerged as an important mechanism in the pathogenesis of AD. Hypoglycemia, possibly as a result of brain insulin resistance leads to decreased O-GlcNAcylation of APP and Tau resulting in their hyperphosphorylation and production on toxic Aβ amyloid and Tau aggregates which are hallmark features of AD. Interestingly, increasing the levels of total O-GlcNAc through highly specific inhibitor of OGA enzyme, Thiamet G has shown promise in the treatment of AD through alleviation of major symptoms of AD through increased O-GlcNAcylation of APP and Tau and reduction in the production of toxic species of these proteins in preclinical studies (Yuzwa et al., 2012; Graham et al., 2014; Hastings et al., 2017). Recent studies have also identified several other proteins with defect in O-GlcNAcylation in AD brain involved in neuronal structure, energy metabolism and insulin signaling. Based on these results, although Thiamet G is considered a potential candidate to be used in the treatment of AD and is likely to be soon in clinics (Figure 1). However, other mechanisms of impaired insulin signaling cascade such as IRS1 and GSK-3 phosphorylation in the pathogenesis of AD as well as other cellular processes need further investigation for development of more effective therapeutic strategy for this devastating neurodegenerative disease.

## Author Contributions

SA and BE read and corrected the manuscript and approved its final content.

## Conflict of Interest Statement

The authors declare that the research was conducted in the absence of any commercial or financial relationships that could be construed as a potential conflict of interest.
